# Predicting Diagnostic Potential of Cathepsin in Epithelial Ovarian Cancer: A Design Validated by Computational, Biophysical and Electrochemical Data

**DOI:** 10.3390/biom12010053

**Published:** 2021-12-30

**Authors:** Hemangi Ranade, Priya Paliwal, Anis Ahmad Chaudhary, Sakshi Piplani, Hassan Ahmed Rudayni, Mohammed Al-Zharani, Ravi Ranjan Niraj, Manali Datta

**Affiliations:** 1 Amity Institute of Biotechnology, Amity University Rajasthan, Jaipur 303002, India; hemangi96.ranade@gmail.com (H.R.); Paliwal.priya5@gmail.com (P.P.); rrkniraj@jpr.amity.edu (R.R.N.); 2 Department of Biology, College of Science, Imam Mohammad Ibn Saud Islamic University, Riyadh 11564, Saudi Arabia; aachaudhary@imamu.edu.sa (A.A.C.); harudayni@imamu.edu.sa (H.A.R.); mmylzarani@imamu.edu.sa (M.A.-Z.); 3 Vaxine Pty Ltd., Flinders University, Bedford Park, SA 5042, Australia; piplani.sakshi@gmail.com

**Keywords:** cathepsin-cystatin, molecular dynamics, differential pulse voltammetry

## Abstract

Background: Epithelial ovarian cancer remains one of the leading variants of gynecological cancer with a high mortality rate. Feasibility and technical competence for screening and detection of epithelial ovarian cancer remain a major obstacle and the development of point of care diagnostics (POCD) may offer a simple solution for monitoring its progression. Cathepsins have been implicated as biomarkers for cancer progression and metastasis; being a protease, it has an inherent tendency to interact with Cystatin C, a cysteine protease inhibitor. This interaction was assessed for designing a POCD module. Methods: A combinatorial approach encompassing computational, biophysical and electron-transfer kinetics has been used to assess this protease-inhibitor interaction. Results: Calculations predicted two cathepsin candidates, Cathepsin K and Cathepsin L based on their binding energies and structural alignment and both predictions were confirmed experimentally. Differential pulse voltammetry was used to verify the potency of Cathepsin K and Cathepsin L interaction with Cystatin C and assess the selectivity and sensitivity of their electrochemical interactions. Electrochemical measurements indicated selectivity for both the ligands, but with increasing concentrations, there was a marked difference in the sensitivity of the detection. Conclusions: This work validated the utility of dry-lab integration in the wet-lab technique to generate leads for the design of electrochemical diagnostics for epithelial ovarian cancer.

## 1. Introduction

Epithelial ovarian cancer (EOC) is the fourth most common cause of cancer-associated death in women in the developing world [1]. The disease typically presents in postmenopausal women, and depicts a lifetime risk of 40–60% [2,3]. CA125 and HE4 have been consistently utilized as clinically approved biomarkers for EOC detection but they have associated advantages and disadvantages [4,5]. Prior detection of EOC increases the life expectancy by 5 years; hence, the availability and utilization of multivariate markers may enable sensitive detection of EOC [6].

**Figure 1:**
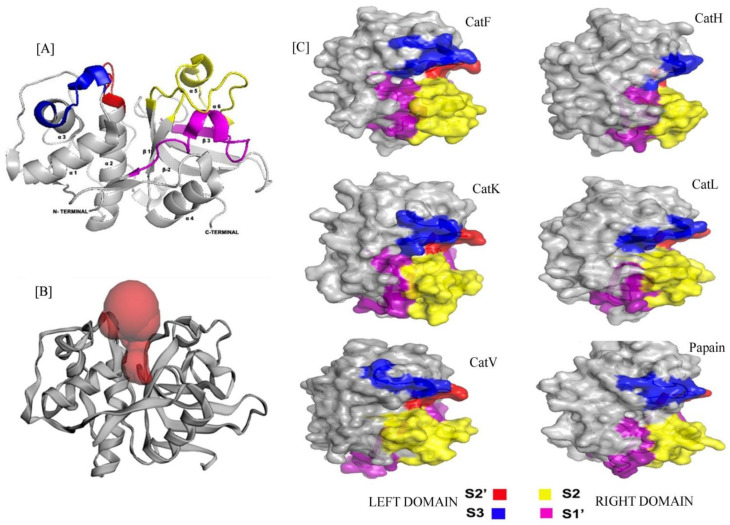
(**A**) Cartoon representation of Cathepsin with Left (L) and Right (R) domains consisting substrate binding subsites S2′ (red), S3 (blue) and S2(yellow), S1′(pink) respectively. The N-terminal L-domain contains mostly α-helix whereas the C-terminal R-domain with mostly β-sheets. The image was prepared by PyMOL. (**B**) The cartoon structure of protein is showing the position of active site cleft at the mid-point of L- and R-domain. The red spheres indicating the presence of binding pocket at this site. The image was created by Computed Atlas of Structure Topography of proteins (CASTp). (**C**) Surface structures of all the cathepsins are showing their structural similarity with papain, comprising S2′(red), S3(blue) substrate binding sites at Left (L) and S2 (yellow), S1′ (pink) substrate binding sites at Right (R) domains. All the images were created by using PyMOL.

Cathepsins are highly expressed in a multitude of human cancers and have been found to be associated with tumor metastasis [7]. This family encompasses cysteine proteases (CPs) (B, C, F, H, L, K, O, S, V, W, X, Z), serine proteases (A and G) and aspartic proteases (D and E) [8]. CPs are ubiquitously expressed, with exceptions being CatK expressed only in osteoclasts and CatV in the thymus and testis [9]. CPs are monomeric proteins with molecular weight ranging between 20 and 35 Kda with their overall structure resembling the native structure of papain. It comprises two functional domains, the N-terminal domain and the C-terminal domain coordinated with a thiolate imidazole ion pair. Amongst the CPs, CatF, CatV, CatL and CatK are endopeptidases, CatC and CatX are exopeptidases, whereas CatH and CatB in addition to their endopeptidase activity possess dual functionality as aminopeptidase and carboxypeptidase [10]. The substrate binding site for cathepsins extends over three well defined subsites, namely S1, S1′and S2 and other subsites like S3 and S4 enabling stabilization of the side chains of the substrates and thus the catalytic prowess of CPs [11,12]. Structural comparison amongst the different CPs indicated a marked demarcation in the substrate binding sites. CatV and CatL exhibited broad specificity for substrate binding at S3 and S1′ sub-sites owing to the sequence variation (Figure 1). In contrast, CatK exhibits substrate specificity at S2 and S2′ with proline as a preferred residue at P2 cleavage position. Comparison of the primary structure of various CPs performed using Clustal Omega indicated conservation of residues at Gly19 of L-domain, residues from Cys22-Trp 26, His 160 and Asn 187 from R-domain. A sequence similarity of almost 79% was found between CatV and CatL, indicating that CatV may be an isoform of CatL.

**Figure 2:**
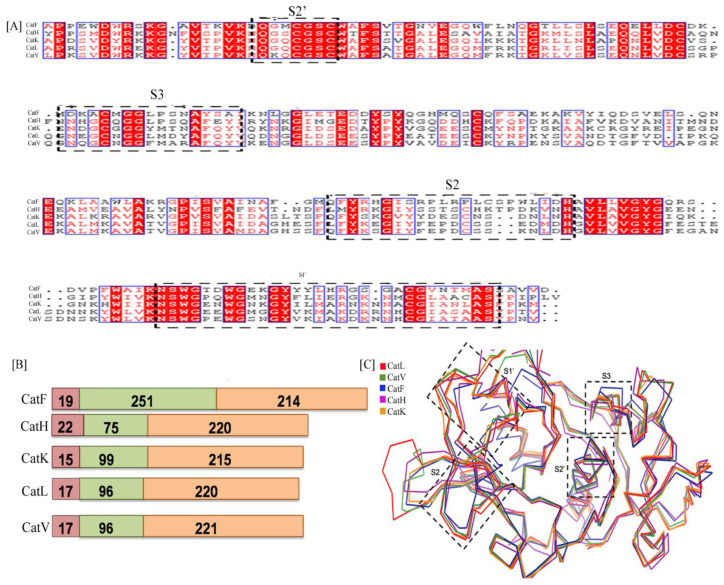
(**A**) The multiple sequence alignment representation of different cathepsins namely CatL (PDB Id. 4AXL), CatK (PDB Id. 6ASH),CatV (PBD Id. 1FH0),CatH (PDB Id. 1NB3), CatF (PDB Id. 1M6D): figure generated by Clustal Omega and Espript3. The substrate binding subsites S2′, S3 of L-domain and S2, S1′of R-domain are highlighted with dotted box. The figure [highlighted in red] denotes sequence conservation of active site residues at their respective subsites. (**B**) The domain architecture of cathepsin indicates conservation in overall functional classification of domains SD represents the signal sequence domain, PD as prodomain, MD is the mature domain; Number of amino acid present in each domain is mentioned. (**C**) Superposition of backbone traces of five cathepsins indicate sequence diversity in domains S1, S2, S3 and S1′ has generated three dimensional perturbations.

Cystatins are protease inhibitors comprising of four classes namely, Type I or Stefin, Type II or Cystatins, Type III or kininogens and Type IV [10,13,14]. Cystatins considered as exosite binding reversible inhibitors of CPs bind adjacent to active sites obstructing the direct access of substrate with the enzyme’s catalytic center [15]. Amongst all Cystatins, a very high interaction with an association rate constant K_ass_ 10^5^–10^7^ M^−1^s^−1^ has been determined for Cystatin C (CysC) and CPs with dissociation equilibrium Kd in the range of 10^−9^–10^−12^ M indicating slow disintegration of the complex [16]. Mature human CysC with a molecular mass of 13Kda possesses three conserved regions namely N-terminal segment, C-terminal interconnected via an L1-L2 loop containing a highly conserved (Q-X-V-X-G) region (Figure 2) [17,18].

The progression of cancer is a multiplex process initiated via an invasion through the epithelial basement membrane progressing to metastasis. The perforation of tumor cells to their neighboring regions is a result of cell-cell, cell-matrix adhesion or degradation and remodeling of extracellular matrix (ECM) [19]. This highly dynamic process of cell-matrix interaction and invasion is known as Epithelial Mesenchymal Transition (EMT). Mimicking natural protein–protein interactions (PPI) may generate reliable partners for some of the disease molecular markers. A large number of such interactions have been discovered and subsequently validated by computational methods and high-throughput experiments. Previous approaches that investigate the information capacity of pre-existing, native networks, our goal with this work is to ask how we can manipulate the biophysics of PPIs to engineer new networks that optimize information transmission. In this article, we have assessed the feasibility of using CysC as a probe for the detection of a class of cancer-specific CPs using computational as well as traditional wet lab experimentation. The CPs selected for this study have been established as components and functionaries enabling EMT with their collagenase and elastolytic activity leading to matrix degradation. [20]. Our study findings may serve as an essential reference for integrating a yet unexplored in vitro protein-protein partnership in the development of electrochromic cancer diagnostics.

## 2. Materials and Methods

### 2.1. Materials

SPMWE-COOH (cMWCNT)/carbon electrode REF: C110CNT (Palmsens, Houten, Netherlands) was purchased from Spain and modified. Cathepsin, Cystatin C (CysC), 1-Ethyl-3-(3-dimethyl-aminopropyl) carbodiimide (EDC) and N-hydroxysuccinimide (NHS) were purchased from Sigma Aldrich, St. Louis, MO, USA. Potassium hexacyanoferrate (III) [K_3_Fe(CN_6_)], Ethanol and other chemicals were obtained from Qualigens, Mumbai India. All other reagents were analytical grade and solutions were prepared in phosphate buffer, pH 7.2 (PB).

### 2.2. PDB File Preparation and Structural Assessment

Structure files for the probable CPs implicated in EOC, namely, CatL, CatK, CatV, CatH, CatF and CysC were downloaded from RCSB-PDB https://www.pdb.org (accessed on 10 October 2021) in .pdb format (Table 1) [21]. Additionally, the amino acid sequence for the CPs and CysC were also retrieved and processed for identification of disordered regions or flexible regions in the proteins using GlobPlot 2.3 http://globplot.embl.de/ (accessed on 10 September 2021) [22] and PONDR-FIT http://www.pondr.com (accessed on 10 September 2021) [23]. The Computed Atlas for Surface Topography of Proteins (CASTp) http://sts.bioe.uic.edu/castp/ (accessed on 10 September 2021) [24] and Consensus Protein-Protein Interaction Site Predictor (Cons-PPISP) https://www.pipe.rcc.fsu.edu (accessed on 10 September 2021) [25] was used to determine the active site of a protein. For docking, only single chains were considered; heteroatoms and water molecules were removed from the PDB files. The heteroatoms found in http://www.pondr.com the proximity of the active site pocket tend to have a substantial impact on the binding of the ligand to the protein. Similarly, water molecules interfere in a similar mechanism, hence both were deleted using the edit command of AutoDock 1.5.6. Energy minimization of the structure was done using SPDBV 4.1.0 [26].

### 2.3. Protein-Protein Docking

The docking was performed using ZDOCK, a protein–protein docking program which performs rigid docking [27] utilizing the Fast Fourier Transform algorithm. Out of the numerous poses generated for CPs and CysC, the top structures were selected based on the ZDOCK’s default scoring function which includes a combination of electrostatics, shape complementarity and statistical potential term. The top ten complexes for each CPs-CysC were selected on the basis of scores and the complexes were assessed for their rotational and translational correctness using PyMol tool [28]. Thereafter using SPDBV 4.1.0, electrostatic potential and molecular surface-based force field energy values were noted, followed by energy minimization using the Steepest Descent method [29]. The docking results were visualized and the hydrophilic and hydrophobic interactions were determined using PyMol 2.4.1 [28].

### 2.4. Molecular Dynamics Simulation

Computer simulations may facilitate understanding of the dynamics of the binding of CatK and CatL with CysC, thus molecular dynamics simulation of the docked protein complex were performed using GROMACS 2018.1 tool [30]. GROMACS 2018.1 is a high-performance molecular dynamics simulation platform that relies on CHARMM36 and SPC/E water model combination to achieve high performance accuracy. The force field Gromos43a1 was selected for the study and protein salvation was executed with SPC water model. The solvated protein was processed for energy minimization using the steepest algorithm up to a maximum of 25,000 steps or until the maximum force (Fmax) is not greater than 1000 kJ/mol nm which is the default threshold. Both complexes CysC-CatK and CysC-CatL were subjected to a 20 ns of final MD simulation followed by intensive analysis in order to characterize many structural and dynamic perturbations. Evaluation of the simulated complexes was done on the basis of root mean square deviation (RMSD) and root mean square fluctuations (RMSF) [31,32].

### 2.5. Electrochemical Assessment of Lead Cathepsins with Cystatin C

To assess the validity of the bioinformatics study, analysis of the shortlisted targets was performed by differential pulse voltammetry (DPV). DPV, a type of electrochemical measurement, is highly sensitive and specific, hence presenting a cost-effective alternative for the design of diagnostic platforms. The experiments were carried out on a Palmsens emstat4 potentiostat controlled by PSTrace5.8 software (Palmsens, Houten, Netherlands). Immobilization of CysC as a probe on a multiwalled screen printed electrode was done as per the protocol cited in Desai et al., 2018 [33]. Briefly, the screen printed cMWCNT electrode (SPMWE) was activated by using 10 mM 1-ethyl-3-(3-dimethylaminopropyl) carbodiimide hydrochloride (EDC) and 10 mM N Hydroxysuccinimide (NHS) (1:1, *v*/*v* in PB) for 1 h followed by washing and drying. Reactive ester groups generated on the working electrode were functionalized by immobilization of CysC. Immobilization of CysC on SPMWE [cMW] was monitored by Faradaic current amplified by the potassium ferricyanide redox mechanism. Thereafter, both candidates, CatL and CatK were incubated on the working area of cMW for 30 min following which DPV measurements were taken. These interactions were tested at physiological conditions (T = 37 °C and pH = 7.4 phosphate-buffered solution) and effect of concentration was also monitored.

## 3. Results

### 3.1. Structural Assessment of Cathepsin Family Members

Disordered regions in the protein have been often implicated in protein interaction and functionality. The disordered regions for the proteins were found using GlobPlot for all selected CPs. The N terminal (1–5) and region comprising of residues 11–29 (Except CatV) were found to be consistently disordered in all the CPs which coincides with the substrate interaction site, S2′.

The major interacting residues which were identified in the S2′ site of CPs are Asn 18, Gln19, Gly20, Cys22, Gly23, Ser24 and Cys25. Another site (54–69), S3 domain implicated in substrate binding was found to be disordered in CATL, CATK and CATF. At S3 site the interacting pocket residues identified by CASTp were Gln60 and Gly68. Both S2′ and S3 site belong to the L domain of the protein structure and are inherently involved in protease-inhibitor interaction sites. The ERRAT plot and PROCHECK tool in SAVES server http://saves.mbi.ucla.edu (accessed on 11 September 2021) enabled us in assessing the overall quality and reliability of the structure files selected for the study whereby the maximum overall quality ranged between 92% and 99% [34,35].

The structure of the available cathepsins were aligned to each other to observe the difference in the three dimensional structure of the candidates. The root mean square deviation (RMSD) values of aligned structures were 0.34 (CatV), 0.39 (CatK), 0.64 (CatF) and 0.76 (CatH) when compared with CatL (Figure 2C).

The S2′ sub-site of L-domain and S1′ of R-domain seems to show an overall conservation in primary as well as their tertiary alignment. The S3 subsite indicates deviations in CatF in the tertiary structure which is justified by the sequence variation in the primary structure (Figure 2A,C) [36].

### 3.2. Interaction of Cystatin C with Different Cathepsins

**Figure 3:**
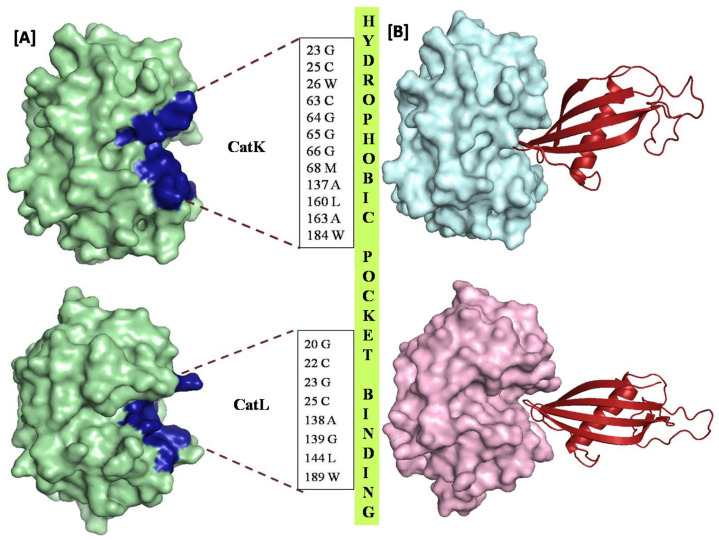
(**A**) Representing the interacting residues of CATK in CYAN and CATL in PALEGREEN with CysC in MAGENTA forming hydrogen bonding network at the binding pocket. H-bonding could be observed between Tyr67 and Val83, Cys25 and Ala84 of CATK and Cys C, respectively, Asp162 and Val83, Trp189 and Trp132 of CATL and CysC respectively. (**B**) Representing the hydrophobic interacting residues of CATK and CATL with CysC. The binding of the CysC in the exosite of CatL is substantiated by the distribution of the hydrophobic moieties in the S2 and S1 subsites, whereas, CatK demonstrates a more uniform distribution the hydrophobic residues in the active site.

Docking is a robust mechanism to assess the affinity and stability of the probe-ligand interactions. The top ten predictions for each of the CPs with CysC were selected and ranked on the basis of predictive free energies. SPDBV was used to determine the free energy of the complexes by force field specification that evaluates energy with partial implementation of the GROMOS96. A more negative value of free energies of the complexes indicated a stronger binding [37]. One pose for each of the CPs, namely, CatL, CatK, CatV, CatH, CatF was selected based on their free energy and subjected to energy minimization in SPDBV to attain a pose with the least inaccurate geometry and minimum energy. On the basis of the energy profiles, two complexes with their respective minimum energy were selected for further analysis, whereby favorable binding partners of CysC were found as CATK and CATL with interaction energies as −16180.323 KJ/mol and −15253.120 KJ/mol, respectively. CysC being an exosite inhibitor does not interact with all the residues of the catalytic triad of CPs consisting of Cys-His-Asn (Figure 3) [18].

Consistent interaction has been observed between CysC and Cys 25 and His 163 (corresponding to CatL sequence). Furthermore, CysC active site residues namely, Gln 81, Ile 82, Val 83, Ala 84, Gly 85; Tyr 128, Pro 131, Trp 132, were found to be interacting with the residues of the CPs located in the S3 and S2′ subsites of the L domain (Figure 3). A summary of contacting residues between the CPs with CysC has been elaborated in Table 1.

CysC was observed to form hydrogen bonding interactions with residues Cys25 and Tyr 67 in CatK whereas in CatL residues implicated were illustrated hydrogen bonding with Asp 162 and Trp 189. The difference in approach of CatK and CatL to CysC lies in the difference in the overall sequence alignment with many dissimilar amino acids found at the interaction sites. The differential interaction patterns are chiefly the reason for different affinities of CPs to CysC. Hydrophobic binding geometry for CatK and CatL by CysC were also analyzed. Comparison of binding of CatK and CatL and their interactions with CysC indicated a predominance of hydrophobic residues located in at the S2′, S2 and S1′ subsite. Analysis of the docked structure indicated that CatL facilitated a ‘snug fit’ for CysC as an exosite binding ligand (Figure 3B), thus justifying the role of hydrophobic contacts.

**Figure 4:**
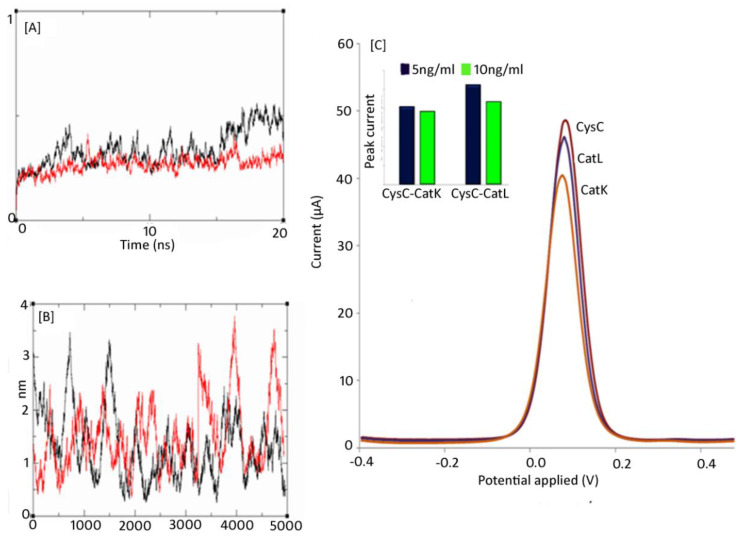
(**A**) Protein RMSD is represented as a function of simulation time in black [CatL] and red [CatK] respectively. (**B**) The per residue RMSF has been represented as a function of the residue number in black [CatL] and red [CatK], respectively, (**C**) The DPV graphs of CysC in the presence of CatL and CatK on the multiwalled carbon nanotube based electrode (inset) Effect of changing concentration of CatL and CatK on peak current response.

The more proficient CATK interaction with CysC may be largely attributed to the uniform distribution of the hydrophobic residues throughout the binding pocket and thus portraying a ‘tighter fit’. The RMSD plot indicated the complexes were stable after commencing the simulation. The RMSD values of CysC-CatK and CysC-CatL seemed to vary between 20−30 Å, thus indicating the ligands have a huge impact on the protein structures. The RMSD plot of CatK was comparatively more stable than for the complete span of 20 ns, suggesting that CatK does not undergo any large conformational changes after binding CysC in comparison to CatL (Figure 4A). Furthermore, CysC-CatL after 17 ns simulation starts showing fluctuations in RMSD in the order of approximately 25 Å, which remained consistent, until the end of simulation time of 20 ns. Moreover, a comparison of the RMSF plot signifies the overall stabilities of CysC-CatK and CysC-CatL complexes. In CatL, the N terminal portion of the protein seems more flexible whereas, in CatK the C-terminal demonstrates more conformational flexibility (Figure 4B). Subsites involved in binding to CysC in CatK and CatL in the complexes exhibited lower confirmational fluctuation, thus justifying the role played by these subsites in the stabilized binding of CysC [32,38,39].

### 3.3. Electrochemical Assessment of the Probe-Biomarker Interaction

In silico interaction analysis generated two positive leads for the diagnosis of ovarian cancer. DPV was performed in presence of 5mM K_3_(FeCN)_6_ in 10 mM KCl as a redox indicator with a potential range of −400 mV to −500 mV with a scan rate of 50 mVs^−1^ [26]. There was a marked fall in the current (51.096 µA for Cys C) when compared with CysC/CatL (46.199 µA) and CysC/CatK (39.5 µA) (Figure 4). Molecular dynamics of the binding interactions confirm the strong affinity of CatK for CysC. As the affinity of CysC-CatK is very high, lesser delocalization of the electrons occurs, resulting in decreased electrochemical detection. Additionally, the residues generating a strong binding interaction in CatK-CysC are hydrophobic in nature. Current generated in DPV studies are due to the crossplay of electrons and their surface concentration in the interacting partners [40]. As a result, electron transfer between the electrode and CatK-CysC became difficult generating a weaker voltametric response. In contrast, the formation of in situ CatL-CysC complexes generated a stronger peak current [41]. CatL interaction with CysC is resultant of electrostatic interaction; signifying a constant exchange of electrons within the species. A constant voltage across the cMW has a tendency to pull the electrons towards the working electrode, which was strongly depicted in CatL-CysC and weak electronic fluctuation in CatK-CysC. The concept was validated upon increasing the concentration of the CatL and CatK showed a significant change in the peak current in CatL but a non-perceptible change upon the increasing concentration of CatK. Upon increasing the concentration of both CatK and CatL, peak current response was more magnified in the case of CatL as compared to CatK (Figure 4C).

## 4. Conclusions

To date, no point of care diagnostic has been developed which may detect ovarian cancer in home settings. Symptoms of cancer may be alleviated if detected early. Hence, a diagnostic device is clearly the need of the hour which may enable regular monitoring of cancer and thus enable timely therapeutic management. Cathepsins have time and again been implicated in pathological manifestations of EOC. Both CatL and Catk have presented themselves as biomarkers for the detection of EOC. The serum level of CatL is significantly higher in patients with malignant EOC and its levels may be used to categorize the severity of the disease. CatK, on the other hand, has been implicated in bone cancer which has the tendency to metastasize to ovaries; hence, the time frame of detection of EOC in case CatK may be subject to the prevalence of cancer in the bones [10]. A diagnostic design was conceptualized for detection of the cathepsin as a biomarker for the screening of asymptomatic EOC candidates. In silico and in vitro experimentation confirmed that both cathepsins, CatL and CatK serve as good candidates for binding to CysC; yet, during electrochemical validation, minimal fluctuations in the concentration of CatL elicited increased difference in peak current and hence an improved sensitivity. As the intended diagnostic prototype is conceptualized on the basis of electrochemical interaction, CatL-CysC presented itself as a more reliable target probe combination. The diagnostic platform designed on the complementary interaction using CatL as a biomarker and CysC as an optimized ligand may supplement prognostic detection of EOC and add to the armamentarium of cancer in vitro diagnostics.

## Figures and Tables

**Table 1:** Table representing the information related to the PDB files used.

PDB ID	Protein Name	Description	Resolution
4AXL	Cathepsin L	Human cathepsin L apo form with ZN	1.92 Å
6ASH	Cathepsin K	Crystal structure of human Cathepsin K with a non-active site inhibitor	1.42 Å
1FH0	Cathepsin F	Crystal structure of human cathepsin F	1.60 Å
1NB3	Cathepsin H	Crystal structure of stefin A in complex with cathepsin H	2.80 Å
1M6D	Cathepsin V	Crystal structure of human cathepsin V complexed with an irreversible vinyl sulfone inhibitor	1.70 Å
3GAX	Cystatin C	Crystal structure of monomeric human cystatin C stabilized against aggregation	1.70 Å

## Data Availability

All data available on online platforms.
